# Novel biomarkers for pulmonary arterial hypertension

**DOI:** 10.1186/s12931-016-0396-6

**Published:** 2016-07-20

**Authors:** Anjum Anwar, Gregoire Ruffenach, Aman Mahajan, Mansoureh Eghbali, Soban Umar

**Affiliations:** Departmentof Anesthesiology, Stanford University, Palo Alto, CA USA; Department of Anesthesiology and Perioperative Medicine, David Geffen School of Medicine at UCLA, Los Angeles, CA 90095 USA

## Abstract

Pulmonary arterial hypertension is a deadly disease characterized by elevated pulmonary arterial pressures leading to right ventricular hypertrophy and failure. The confirmatory gold standard test is the invasive right heart catheterization. The disease course is monitored by pulmonary artery systolic pressure measurement via transthoracic echocardiography. A simple non-invasive test to frequently monitor the patients is much needed. Search for a novel biomarker that can be detected by a simple test is ongoing and many different options are being studied. Here we review some of the new and unique pre-clinical options for potential pulmonary hypertension biomarkers. These biomarkers can be broadly categorized based on their association with endothelial cell dysfunction, inflammation, epigenetics, cardiac function, oxidative stress, metabolism,extracellular matrix, and volatile compounds in exhaled breath condensate. A biomarker that can be detected in blood, urine or breath condensate and correlates with disease severity, progression and response to therapy may result in significant cost reduction and improved patient outcomes.

## Background

### Pulmonary arterial hypertension

Pulmonary arterial hypertension (PAH) is a rare yet deadly disease with many causes ranging from idiopathic to secondary to many underlying pathologies including congenital heart diseases. The disease course is monitored by pulmonary artery systolic pressure measurement via transthoracic echocardiography. The confirmatory and gold standard is the right heart catheterization. This invasive test is used for diagnosis as well as for prognosis of the disease. For obvious reasons, a non-invasive test to monitor patients frequently will be invaluable. Therefore, the search for a biomarker that can be detected by a simple blood test is ongoing, and many different options are being studied. This literature review outlines some of the selected novel candidates for a potential biomarker for diagnosis and prognosis of PAH. These biomarkers are broadly categorized based on their relationship to endothelial cell dysfunction, inflammation, epigenetics, cardiac function, oxidative stress, metabolism, extracellular matrix, and volatile compounds (Table 1).

**Table 1:** A comprehensive table showing the different groups of biomarkers discussed in this review along with the number of subjects, major findings and limitations of the individual studies

	Biomarkers	Subjects	Major finding/limitations
Endothelial cell Markers	ADMA	PAH (35)	Increased serum concentration in PAH correlated significantly with mPAP and PVR [54].
		CTR (35)	
		PAH-CHD (30)	No correlation between the significantly increased ADMA concentration in PAHCHD patients and hemodynamic parameters [39].
		CHD (20)	
		CTR (20)	
	Circulating angiogenic modulatory factors	PAH (97)	Serum concentrations of sEng, sVEGFR1 and CRP were all elevated in PAH patients with sEng having the greatest predictive value for PAH. sEng and CRP were determined to be the most sensitive independent markers for predicting survival [25].
		CTR (56)	
	PCEB-ACE	iPAH (25)	These new techniques allow for the assessment of endothelial function in vivo, but these are currently only for research purposes [32].
		PAH-CTD (19)	
		CTR (23)	
Inflammatory markers	GDF-15	PAH-naïve (76)	Significant upregulation in PAH correlated with mean RAP. Plasma concentrations > 1200 ng.L-1 increased the risk of death [30].
		PAH-treated (22)	
	Galectin-3	PAH (15)	Significantly increased in serum of PAH patients which correlated with RV hemodynamics [8].
		CTR (10)	
	OPN	iPAH (70)	Increased plasma concentration in PAH correlated with NYHA functional class. 4 year survival rate increased in concentrations < 34.5 ng.mL-1 [23].
		CTR (40)	
	MIF	Ssc-PAH (15)	Circulating concentration is significantly increased in Ssc-PAH vs. Ssc and correlated with NYHA functional class [44].
		Ssc (14)	
	NLR	PAH (*n* = 101)	Significant correlation with NYHA functional class and mortality; but not an independent predictor of mortality [34].
	Serum BCL-2	Child-PH (35)	Increased sBCL-2 concentration correlated with NYHA functional class [1].
		CTR (38)	
	miR	iPAH (12)	Lists circulating miR that are differentially expressed in PAH vs. control [40].
		CTR (10)	
Heart function	BNP/NTproBNP and RDW	iPAH (139)	Plasma NT-proBNP is significantly correlated with PVR, CI, and mean RAP. RDW predicted survival independently from NTproBNP and the 6MWD test [37].
		CTR (40)	
	Cystatin C	PAH (14)	Significantly correlates with cardiac and hemodynamic parameters [7].
		CTR (10)	
	Homocysteine	PAH-CHD (30)	Significant increasein PAH-CHD compared to other groups but no hemodynamic correlation was found [39].
		CHD (20)	
		CTR (20)	
	Cardiac troponin	PAH (167)	Increased concentration of cardiac troponin correlates with mortality [48].
New cardiovascular biomarkers	MRproADM	HF (728)	Significant correlation between increased plasma concentration of MRproADM and mortality [15].
	CT-proET1	PAH (28)	Blood concentration increased with WHO functional class and correlated with mortality [43].
	Copeptin	PAH (92)	Expression correlated with survival and disease severity [43].
		dCTR (39)	
		hCTR (14)	
Oxidative stress	F2-Isoprostanes	PAH (110)	Patients with urine concentration above the median have an increased risk of death [4].
	Oxidized lipids	PAH (28)	Significantly increased in serum of PAH patients vs. control [38].
		CTR (21)	
	GSH	PAH (12)	Significantly decreased in plasma of PAH patients [54].
		CTR (12)	
Metabolic biomarkers	Fisher ratio	PH (140)	Decreases with PH severity. Most measured amino acid levels were significantly higher in patients with PAH [52].
		CTR (140)	
	Tryptophan metabolites	PAH (35)	Anthranilate and Quinolinate were increased with PVR above 2 [21].
		CTR (36)	
	Gherlin	PAH (20)	Plasma levels were significantly increased in PAH and correlated with PASP [53].
		CTR (20)	
ECM	MMP-2 TIMP-4	PAH (36)	All three proteins were significantly increased in plasma of PAH patients but did not distinguish between functional classes [41].
	TNC	CTR (44)	
Volatile compounds		PAH (27)	May allow the detection of PAH specific volatile compounds [26].
		CTR (30)	

### Overview of a selection of novel biomarkers for pulmonary hypertension

#### Endothelial cell markers

##### Asymmetric dimethylarginine (ADMA)

ADMA is a natural amino acid and endogenous inhibitor of nitric oxide (NO) production. Its role has been studied in cardiovascular diseases for many years. ADMA is generated from methylation of arginine residues by protein arginine methyltransferases and subsequent proteolysis, while its elimination is achieved mainly by degradation with dimethyl arginine dimethyl aminohydrolase. The research by Zhang et al. showed that endothelial injury can cause increase in plasma ADMA concentration. The ADMA concentrations are associated with reduced production of NO, affecting NO/cGMP pathway and thus leading to increase in vascular tone. The authors contended that the antioxidative potential is decreased and ADMA concentrations can be a useful biomarker for PAH progression [54].

In an earlier study, Giannakoulas et al., showed that ADMA may induce pulmonary endothelial dysfunction via changes in the expression and activity of connexin 43 [9]. Connexins are transmembrane proteins that form hemichannels and gap junctions on the cell membranes, and are involved with the transfer of small signaling molecules between the cytoplasm and extracellular space and between connecting cells. Among connexins, suppressing connexin 43 expression or function promotes skin wound closure and granulation tissue formation, and may alleviate scarring, but the mechanisms are not well understood. How ADMA affects connexins is yet to be studied.

In a study by Sanli et al., the authors studied the concentrations of ADMA in pediatric patients with congenital heart disease. The concentrations were elevated in patients with PAH and congenital heart disease and they made the recommendations for large prospective clinical studies before establishing it as a biomarker [39].

##### Circulating angiogenic modulatory factors

Circulating angiogenic modulatory factors, which are believed to regulate angiogenesis, are also being studied for their role as biomarkers for patients with PAH. Malhotra et al. investigated the concentrations of soluble endoglin, soluble vascular endothelial growth factor receptor-1 (sVEGFR1), N-terminal brain natriuretic peptide (NTproBNP), C-reactive protein (CRP), and other biomarkers in peripheral blood in 97 patients with PAH. They found that circulating angiogenic proteins like soluble endoglin and sVEGFR1 concentrations were increased. They also obtained lung tissue from patients and studied the endoglin expression in microvascular endothelium and found that the expression was significantly enhanced [25]. Tiede et al. confirmed these results in a study with 76 PAH patients at the time of diagnosis, but they didn’t find a correlation between sVEGFR1 or placental growth factor and haemodynamic parameters, 6-min walk distance or survival [46]. Al-Naamani et al. recently showed that lower von Willebrand factor activity at baseline was associated with an increased risk of death or lung transplantation in patients with PAH [2].

Damico et al. investigated the relationship of a potent angiostatic factor, endostatin, with disease severity and mortality in PAH. Serum endostatin correlated with poor functional status, decreased exercise tolerance, and invasive hemodynamic variables. Furthermore, serum endostatin was a strong predictor of mortality [5]. Another study demonstrated the usefulness of circulating angiopoietins as biomarkers. In this study Kumpers et al. demonstrated that angiopoietin 2 blood concentration correlates with pulmonary vascular resistance making it an accurate survival predictor [16].

##### Pulmonary capillary endothelium-bound angiotensin converting enzyme (PCEB-ACE)

PCEB-ACE activity has been recently highlighted as a marker of pulmonary endothelial function in several diseases including idiopathic PAH, PAH associated with connective tissue disease and in systemic scleroderma without any sign of PAH [17, 32]. Moreover, Langleben et al. recently demonstrated that during a challenge with epoprostenol, patients who didn’t respond to the treatment had a decreased activity of PCEB-ACE. Conversely, PCEB-ACE activity did not decrease in responding patients [18].

#### Inflammatory markers

##### Growth Differentiation Factor-15 (GDF-15)

GDF-15 is a murine transforming growth factor β superfamily member and highly expressed in the adult liver. It is a stress responsive cytokine and plays a role in regulating inflammatory pathways during a disease and tissue injury. Nicket et al., showed that GDF-15 concentrations are elevated in patients with tissue hypoxia, acute coronary syndrome, pulmonary embolism and in patients with idiopathic PAH (iPAH). This increase in GDF-15 concentrations may be explained by the involvement of multiple stress pathways in cardiac tissue injury and, therefore, can be utilized as an indicator of disease prognosis [30]. The authors found that GDF-15 enhances the prognostic information provided by more established biomarkers, including NTproBNP, as measured by clinical indicators like six-minute walk distance. However, GDF-15 is not a cardiac specific cytokine, so its use as an independent biomarker is questionable. More studies are needed before it can be established as a prognostic marker.

##### Galectin-3

Galectin-3 is secreted by macrophages in response to mechanical and neurohormonal stimuli. It works as an endocrine and paracrine factor to stimulate other macrophages, fibroblasts, and inflammatory cells. There is evidence for its significance in patients with heart failure [12]. This is based on its increased concentrations in patients with left heart failure where it is believed to play a major role in cardiac remodeling and fibrosis. Recently Fenster et al. studied the concentrations of galectin-3 in patients with RV dysfunction and PAH. They performed echocardiography and measured blood concentrations of galectin-3 on the same day in 15 patients with PAH and found a significant correlation between RV morphological changes and galectin-3 concentrations. Galectin-3 concentrations are elevated in all patients with PAH [8]. Galectin-3 has a limited role in PAH when used as a sole biomarker. Its concentrations are elevated in patients diagnosed with other diseases including renal failure and pulmonary and hepatic fibrosis [22]. Nonetheless, its use could be of interest in combination with others biomarkers.

##### Osteopontin (OPN)

OPN is another cytokine that is increased when inflammatory or neoplastic processes activate the signaling pathways. OPN also activates inflammatory cells including macrophages, macrocytes, and lymphocytes.

Our lab observed increased circulating plasma OPN concentrations in rats with Monocrotaline-induced PAH that responded to therapy [28]. This finding was validated in patients by Lorenzen et al. Their study concluded that patients with iPAH had increased OPN concentrations. Circulating OPN concentrations were especially helpful in monitoring patient response to therapeutic intervention [23]. Lorenzen et al. showed that OPN correlated with exercise capacity and functional class and was an independent predictor of survival. Since elevated OPN concentrations are not specific to iPAH, more studies are needed to establish its benefits as prognostic biomarker in patients with iPAH.

##### Macrophage migration inhibitory factor (MIF)

In a recent study, Le Hiress et al. found that concentrations of circulating MIF were increased in the serum of patients with PAH compared to control subjects, and it implicated T-cell lymphocytes as the source of this overabundance. Furthermore, they found that curative treatments with the MIF antagonist ISO-1 or anti-CD74 neutralizing antibodies partially reversed the development of PH in rats and substantially reduced inflammatory cell infiltration [19].

Stefanantoni et al., recently found significantly higher circulating concentrations of MIF and stem cell growth factor β (SCGF β) in patients with iPAH and with PAH secondary to systemic sclerosis. Higher concentrations of MIF were found in patients with an higher NYHA class– indicating that MIF could be used as a prognostic marker [44].

##### Neutrophil-to-lymphocyte ratio (NLR)

In a clinical study, Özpelit et al., recently discovered that NLR was correlated with well-established prognostic markers in PAH such as NYHA functional class, BNP concentrations, and tricuspid annular plane systolic excursion [34].

##### Serum BCL-2 (sBcl-2)

Akin et al., assessed concentrations of sBcl-2 in children with PH and reported that there was an increase in the concentrations of sBcl-2 as an inflammatory marker and that the sBcl-2 concentrations are associated with prognostic parameters in children with PH [1].

##### Chemokine CXC ligand 13 (CXCL13)

Chemokine CXC ligand 13 (CXCL13) has been implicated in perivascular inflammation and pulmonary vascular remodeling in patients with IPAH. Olsson et al. investigated whether CXCL13 may also play a role in chronic thromboembolic pulmonary hypertension (CTEPH) and whether serum levels of CXCL13 might serve as biomarkers in IPAH and CTEPH. CXCL13 was overexpressed in pulmonary vascular lesions of patients with IPAH and CTEPH, and increased serum concentrations were found in patients with IPAH and CTEPH, suggesting a potential pathogenic role of CXCL13 in both diseases. The authors concluded that given the weak associations between serum CXCL13 and markers of disease severity and outcome, CXCL13 is unlikely to become a promising biomarker in these patient populations [31].

#### MicroRNAs as biomarkers in PAH

MicroRNAs (miRNAs) are small non-protein coding genes, which function in RNA silencing and post-transcriptional regulation of gene expression. miRNAs circulate in the blood, and their concentrations can be a reflection of existing vascular pathology.

Sarrion et al., demonstrated the role of miR23a, miR-130, miR-191, miR-204, miR-145, miR-27a, miR-328, miR-1-2, miR-199, and miR-744 as potential biomarkers of iPAH. Out of these, miR-23a is of special interest because of its relation to pulmonary function. It is also believed to regulate the transcription of genes implicated in PH disease progression including peroxisome proliferation-activated receptor (PPAR) γ coactivator-1α (PGC1α), which is currently being studied as a potential biomarker for PH progression [40]. In a rat model of PH, Paulin et al. found that serum tumor necrosis factor-α concentrations progressively increased with time while serum miR-208 concentrations decreased which mirrored its concentrations in the diseased RV myocardium [35].

#### Heart function related biomarkers

##### B-type natriuretic peptide (BNP) and amino-terminal pro-B-type natriuretic peptide (NTproBNP)

BNP and NTproBNP are the only biomarkers recommended by the current guidelines for risk stratification in PAH. However only BNP is cited in guidelines addressing PAH treatment endpoints: a “normal” BNP is suggested as a treatment goal. This is true for both adults and pediatric patients. The relevance of BNP and NTproBNP as markers of disease progression are known since almost 20 years for IPAH and associated PAH [29, 51]. A retrospective study done by Takatsuki et al., studied 88 children with PH found that BNP and NTproBNP are strong predictors of disease progression and mortality. However BNP correlates better with the hemodynamic changes because of its shorter half-life and NTproBNP is a better mortality predictor [45]. NTproBNP concentration is not only a good predictor of survival in PAH, but also a good marker of treatment efficacy. Indeed, Andreassen et al. demonstrated a decrease of NTproBNP concentration in PAH patients responding to therapy [3]. Furthermore, a retrospective study demonstrated that a NTproBNP concentration ≥1256 pg.mL^-1^ at the time of diagnosis was a predictor of poor outcome. Also the same study highlighted the fact that a decrease of 15 % of NTproBNP concentration was associated with survival in their patient’s cohort [27]. Al-Naamani et al. recently showed that lower vWF activity and cholesterol concentrations and higher NTproBNP concentrations at baseline were associated with an increased risk of death or lung transplantation in patients with PAH [2].

##### Red Cell Distribution Width (RDW)

RDW has been studied as a biomarker for outcomes in patients with cardiovascular diseases [6, 47]. Its relation with carotid atherosclerosis in hypertension has been explained [50]. RDW is being studied as another potential biomarker for measurement of prognosis in patients with iPAH. In a study by Rhodes et al., the authors studied 139 patients with iPAH. Their approach was to study multiple biomarkers and focus on RDW, GDF-15, IL-6, creatinine and NTproBNP. They measured these biomarkers in the patients and found that their concentrations were related to the disease severity. Additionally, the authors found that if RDW is measured along with NTproBNP, it can significantly help in disease severity measurement and can potentially be a significant prognosis indicator [37].

##### Cystatin C

Cystatin C has been studied as a potential biomarker in patients with PAH. It is used as an indicator for renal filtration and is measured in patients with renal insufficiency. Fenster et al. studied the correlation of serum cystatin C concentrations and anatomical and physiological parameters of right ventricular function. They found abnormally high concentrations in patients with PAH which correlated with right ventricular function [7]. While the study sample was small, and included ten patients and controls, the cystatin C concentrations were significantly elevated in patients with PAH. Cystatin C can be used along with the BNP and NTproBNP and is independent of age, gender or muscle mass making it a potentially more desirable biomarker in PAH.

##### Elevated homocysteine

In a study by Sanli et al., the authors studied the relationship of homocysteine and ADMA concentrations in patients with congenital heart disease. They found increased concentrations in patients having PAH and congenital heart disease but no correlation was found between hemodynamic parameters and homocysteine concentration. They also found that this increase in concentration was more significant in cyanotic patients as compared to the ones without cyanosis [39]. In another study done in patients with CHD, Ozerol et al. also revealed increased homocysteine concentrations in patients with CHD and PAH as compared to patients who had left to right shunt surgery and no PAH [33]. However still a large-scale clinical study is required to recommend homocysteine as a biomarker for diagnosis and prognosis of PAH.

##### Cardiac troponin I

Velez-Martinez et al. reported cardiac troponin I concentrations, detectable with a novel highly sensitive assay, in PAH patients with more severe hemodynamic and cardiac structural abnormalities [48]. Cardiac troponin I levels, detectable with this novel highly sensitive assay, identify patients with PAH who have more severe hemodynamic and cardiac structural abnormalities and provide novel and independent prognostic information. This highly sensitive assay has the potential to detect more at-risk patients and improve current risk-stratification algorithms.

##### New cardiovascular biomarkers

During recent years, the new cardiovascular biomarkers mid-regional pro-adrenomedullin (MR-proADM), mid-regional proatrial natriuretic peptide (MR-proANP), Copeptin, and carboxy-terminal pro-endothelin-1 (CT-proET1) have been suggested to predict prognosis and aid clinical decision-making in various cardiopulmonary diseases [13, 15, 24, 43]. Kolditz et al., recently studied the correlation between biomarkers and hemodynamic and exercise parameters in PAH patients. They concluded that different biomarkers reflect distinctive disease aspects in PAH. NTproBNP best predicts hemodynamic impairment while MR-proADM strongly correlates with exercise capacity [14]. In an interesting study, Volkers et al., demonstrated the increase of myocardial high-sensitive Troponin T in patients with PAH in response to maximal physical exercise while concentrations of other biomarkers remained constant after exercise testing [49]. Heresi et al. demonstrated that low plasma high-density lipoprotein cholesterol (HDL-C) is associated with higher mortality and clinical worsening in PAH [10]. Zhao et al. demonstrated that serum HDL cholesterol levels might serve as an indicator of disease severity and prognosis in patients with IPAH [55].

#### Oxidative stress related biomarkers

Inflammation and oxidative stress are essential in PAH with increased lipid peroxidation and reduced antioxidant defenses [36].

##### F2-Isoprostanes

The hunt for biomarkers for iPAH is not limited to blood and serum. A few studies have looked for biomarkers in other biofluids. One of these studies was done by Cracowski et al. prospectively. They studied a compound called F2-isoprostanes in urine samples of 110 adults. This compound is considered as a marker of oxidative stress and its exact source has not been confirmed yet. They found that F2-isoprostane is an independent predictor of three-year mortality in patients with iPAH. They also concluded that measurement of this compound in urinary samples of asymptomatic children with family history of iPAH can help in early detection of disease process [4].

##### Oxidized lipids

Oxidized lipids such as hydroxyeicosatetraenoic (HETE) and hydroxyoctadecadienoic acids (HODE) have an established role in the pathogenesis of vascular diseases. Recently, the research in our lab showed that miR193 overexpression can lead to a significant improvement in the severity of pulmonary hypertension [42]. We studied two rodent models of PH and discovered that plasma concentrations of oxidized lipids such as HETEs and HODEs were significantly elevated in PH. MicroRNA analysis revealed that miR193 was significantly downregulated in the lung tissue and serum from both patients with PAH and rodents with PH. Apolipoprotein A-I mimetic peptide 4 F treatment reduced the concentrations of oxidized lipids and rescued preexisting PH in both rodent models through miR193 overexpression in the lungs. We concluded that A-I mimetic peptide 4 F and microRNA-193-3p may have therapeutic implications in patients with PAH. Furthermore, oxidized lipids may serve as plasma biomarkers for PAH. Recently, we and our collaborators at UCLA found significantly increased plasma concentrations of the eicosanoids 9-HODE, 13-HODE, 5-HETE, 12-HETE, and 15-HETE in patients with PAH [38]. The study has further implicated the putative role of oxidative stress and inflammation in the pathobiology of PAH.

##### Other oxidative stress related markers

Reis et al., looked at oxidative-stress biomarkers in patients suffering from PAH and found that inflammation and oxidative stress were present in patients with PAH, as confirmed by increased lipid peroxidation, reduced GSH, and low concentrations of vitamin E [36].

#### Metabolic biomarkers

##### Circulating amino acid profile and fischer ratio

Plasma amino acid concentrations are being studied in an effort to look for a biomarker in patients with PAH. In a study by Yanagisawa et al., distinct patterns of amino acids (aminogram) of 140 patients with PAH were studied [52]. The authors also measured the Fischer ratio (branched-chain amino acids/aromatic amino acids). The study concluded that patients with PAH have a recognizably different aminogram and Fischer ratio was decreased with increasing severity of disease. However this study has a few limitations and in order to confirm aminogram as a potential biomarker for PAH a large-scale investigation is needed. Some of the major limitations of this study include the patients with PH receiving PH-specific drugs and diuretics; that may have augmented the differences in amino acid profile and Fischer ratios. In addition, NYHA functional class and gender were not evenly distributed among the patients with PH and the majority of this study population comprised patients with CTEPH, and thus, the results of this study in all enrolled patients might have been affected or masked by multiple etiologic conditions. Furthermore, the absolute change in amino acid levels between PH and healthy control subjects was generally small although statistically significant.

##### Metabolomics related markers

Metabolomics involves study of the metabolic activities and their end products or metabolites at the cellular concentration. As all the disease processes start with the malfunction at the cellular concentration including the oxidative response, metabolomics can provide a breakthrough for diagnosis and prognosis of the medical problems. A few studies have been performed to study the metabolic profiling and its relation with the cardiovascular diseases including PAH. The metabolites of interest included all from simple lipids, glucose to small molecules like cGMP. There is still a need to perform large-scale studies to detect a particular role of metabolic profiling in diagnosis and prognosis of diseases. The study by Lewis et al. showed that metabolomics along with high-throughput genome sequencing and RNA expression analysis can also be helpful to discover more biomarkers of great interest for PAH and RV dysfunction [20]. Recently, Lewis et al. sought to determine whether metabolite profiling could identify plasma signatures of right ventricular-pulmonary vascular (RV-PV) dysfunction. They measured plasma concentrations of 105 metabolites using targeted mass spectrometry in 71 individuals. Metabolic profiling identified reproducible signatures of RV-PV dysfunction, highlighting both new biomarkers and pathways for further functional characterization [21].

##### Tryptophan metabolites

In a recent study, Lewis et al. Showed that right ventricular-pulmonary vascular hemodynamic parameters correlate strongly with a subset of tryptophan metabolites. A strong correlation was found with the concentrations of the 4 tryptophan metabolites of the indoleamine 2,3-dioxygenase pathway: kynurenine, kynurenate, anthranilate, and quinolinate [21].

##### Ghrelin

Yang et al. found that plasma ghrelin concentrations were elevated in IPAH patients. Ghrelin is a peptide hormone produced in the gastrointestinal tract and regulates appetite. Increased ghrelin concentrations correlated positively with right ventricle diameter, pulmonary arterial systolic pressure, plasma N-terminal brain natriuretic peptide, endothelin-1 and nitric oxide concentrations [53].

#### Extracellular matrix related biomarkers

Schumann et al. in 2010 tested the hypothesis that plasma concentration of matrix metalloproteinase (MMP)-2, tissue inhibitor of matrix metalloproteinases (TIMP)-4 and tenascin C (TNC) might be useful as biomarkers for assessing the severity of PH. Therefore, the concentrations of MMP-2, TIMP-4, TNC and NTproBNP of 36 PH patients were compared with those of 44 age- and gender-matched healthy volunteers. Interestingly, in PH patients, significantly elevated plasma concentrations of MMP-2, TIMP-4, TNC and NTproBNP were detected. In particular, TIMP-4 was significantly increased in patients with higher NYHA classification, and in patients with severe right ventricular hypertrophy [41]. Hessel et al., in 2009 found that Monocrotaline-induced PH and RV failure was associated with an upregulation of myocardial Tenascin C gene expression, resulting in re-expression of myocardial Tenascin C protein concentrations, and elevated Tenascin C plasma concentrations. As RV ejection fraction correlated significantly with Tenascin C plasma concentrations, they suggested plasma concentrations of Tenascin C may serve as a marker of PH-induced RV failure [11].

#### Volatile compounds in exhaled breath condensate in PAH

Mansoor et al., compared exhaled breath condensate (EBC) samples from 30 age-matched normal healthy individuals and 27 NYHA class III and IV IPAH patients and analyzed volatile organic compounds in EBC samples using gas chromatography/mass spectrometry. There were 62 unique compounds in the control group, 32 unique compounds in the IPAH group, and 14 in-common compounds between groups. Six compounds significantly correlated with pulmonary hemodynamic variables such as mPAP, PVR or PAWP important in IPAH diagnosis [26].

## Conclusions

**Fig. 1:**
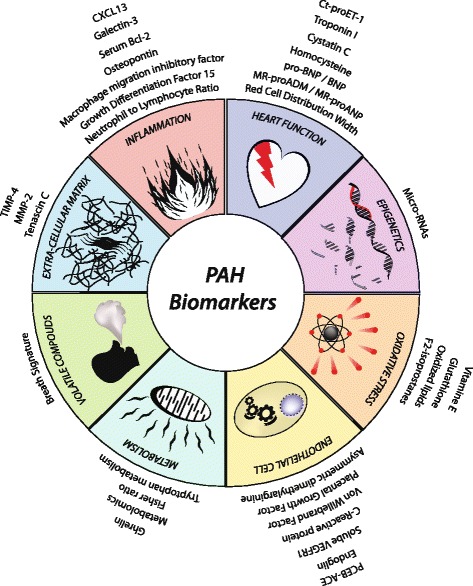
Schematic diagram showing different groups of biomarkers for pulmonary hypertension including markers related to inflammation, heart function, epigenetics, oxidative stress, endothelial function, metabolism, volatile compounds, and extracellular matrix

The search for a non-invasive biomarker for pulmonary hypertension is ongoing. Although a number of promising pre-clinical and clinical biomarkers for PAH have been mentioned in this review, the challenges of validation and correlation with hemodynamic parameters remain (Fig. 1). Large clinical studies are needed to validate the promising biomarkers. In addition, more sensitive and specific assays will help in validation of the biomarkers. It is likely that a single biomarker may not provide all the relevant information required for an individual patient, and the potential for a biomarker panel is of considerable interest. There is a possibility of discovering biomarkers for PAH corresponding to various compartments within the lung such as the pulmonary vasculature, inflammatory infiltrate, and extracellular matrix or biomarkers related to RV function. Biomarker/s that can be detected by a simple blood test or breath condensate and correlate/s with disease severity, progression and response to therapy may result in significant cost reduction and improved patient outcomes.
